# Forsythoside A Controls Influenza A Virus Infection and Improves the Prognosis by Inhibiting Virus Replication in Mice

**DOI:** 10.3390/molecules21050524

**Published:** 2016-04-26

**Authors:** Li Deng, Peng Pang, Ke Zheng, Jiao Nie, Huachong Xu, Sizhi Wu, Jia Chen, Xiaoyin Chen

**Affiliations:** Department of Traditional Chinese Medicine, School of Medicine, Jinan University, Guangzhou 510632, China; pangpeng@stu2014.jnu.edu.cn (P.P.); zhengke@stu2014.jnu.edu.cn (K.Z.); 18819351723@163.com (J.N.); xuhuachong@stu2015.jnu.edu.cn (H.X.); wusizhi@stu2015.jnu.edu.cn (S.W.); fredy212@foxmail.com (J.C.)

**Keywords:** forsythoside A, influenza A virus infection, TLR7 signaling pathway, oseltamivir

## Abstract

*Objective*: The objective of this study was to observe the effects of forsythoside A on controlling influenza A virus (IAV) infection and improving the prognosis of IAV infection. *Methods*: Forty-eight SPF C57BL/6j mice were randomly divided into the following four groups: Group A: normal control group (*normal con*); Group B: IAV control group (*V con*); Group C: IAV+ oseltamivir treatment group (*V oseltamivir*; 0.78 mg/mL, 0.2 mL/mouse/day); Group D: IAV+ forsythoside A treatment group (*V FTA*; 2 μg/mL, 0.2 mL/mouse/day). Real-time fluorescence quantitative PCR (RT-qPCR) was used to measure mRNA expression of the TLR7, MyD88, TRAF6, IRAK4 and NF-κB p65 mRNA in TLR7 signaling pathway and the virus replication level in lung. Western blot was used to measure TLR7, MyD88 and NF-κB p65 protein. Flow cytometry was used to detect the proportion of the T cell subsets Th1/Th2 and Th17/Treg. *Results*: The body weight began to decrease after IAV infection, while FTA and oseltamivir could reduce the rate of body weight loss. The pathological damages in the FTA and oseltamivir group were less serious. TLR7, MyD88, TRAF6, IRAK4 and NF-κB p65 mRNA were up-regulated after virus infection (*p* < 0.01) while down-regulated after oseltamivir and FTA treatment (*p* < 0.01). The results of TLR7, MyD88 and NF-κB p65 protein consisted with correlative mRNA. Flow cytometry showed the Th1/Th2 differentiated towards Th2, and the Th17/Treg cells differentiated towards Treg after FTA treatment. *Conclusions*: Our study suggests forsythoside A can control influenza A virus infection and improve the prognosis of IAV infection by inhibiting influenza A virus replication.

## 1. Introduction

Influenza infections represent a considerable public health burden. Each year, influenza viruses infect 3 to 5 million people worldwide [1,2]. Influenza illness is characterized by involvement of the respiratory tract accompanied by systemic complaints, including headache, myalgia, and fever [3]. After influenza virus infection, a rapid immune response is required to control the lung infection. The role of innate immunity against influenza virus has been widely demonstrated to be essential of defense against viral infections through transmembrane cell receptors [4]. Toll-like receptor 7 (TLR7) is one of these receptors which can recognize viral single-stranded RNA (ssRNA) [5], then activate the downstream signaling molecules through the MyD88-dependent pathway. MyD88 activation leads to the death domain of interleukin-1 receptor-associated kinase (IRAK1) and tumor necrosis factor receptor-associated factor 6 (TRAF6) activation in sequence.

**Figure 1:**
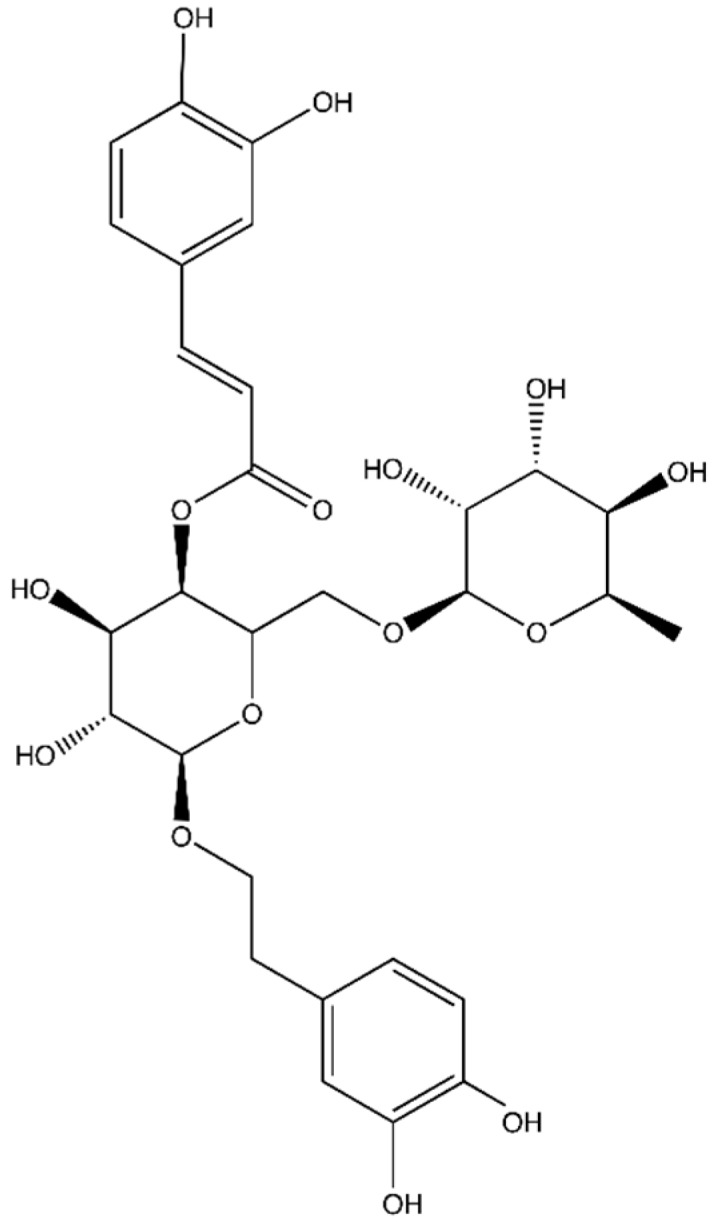
Chemical structure of forsythoside A.

In Asia medicinal herbs have been used for many centuries. It’s interesting that plants, including *Forsythia suspense* fruits, show remarkable results in treating influenza [6]. Forsythoside A (FTA) is one of the main phenylethanoid glycosides from *Forsythia suspense* (Figure 1). Research was presented showing that FTA had antimicrobial activity and antivirus activity [7,8,9]. But whether FTA can treat IAV infection, and if it could, little is known regarding the mechanism of FTA in treating influenza.

Based on the fact that the TLR7 signaling pathway is initially involved in microbial recognition by the immune system, and the antimicrobial activity and antivirus activity of FTA, the present study was designed to test the hypothesis that FTA could exert effects on the regulation of the TLR7 signaling pathway after respiratory tract IAV infection.

## 2. Results 

### 2.1. Changes in Body Weight

**Figure 2:**
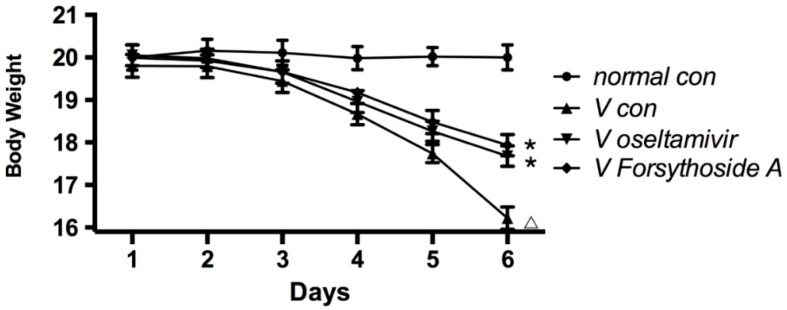
Changes in mice body weight. The body weight of mice in *V con*, *V oseltamivir* and *V FTA* began to decline on day 3. Body weight of Mice in *V* *con* decreased more quickly than *V oseltamivir* and *V FTA*. Δ *p* < 0.01 compared with *normal con*. * *p* < 0.01 compared with *V con*.

The body weight changes of the mice in different experimental groups are shown in Figure 2. Body weight of mice in *normal con* showed no changes. After IAV infection, the physical condition of the mice in the *V con*, *V oseltamivir* and *V FTA* groups started to deteriorate, and they began to lose body weight. The weight of the *V con* animals decreased more quickly. There were no statistical differences between *V oseltamivir* and *V FTA*. 

### 2.2. Changes in Lung Tissue

**Figure 3:**
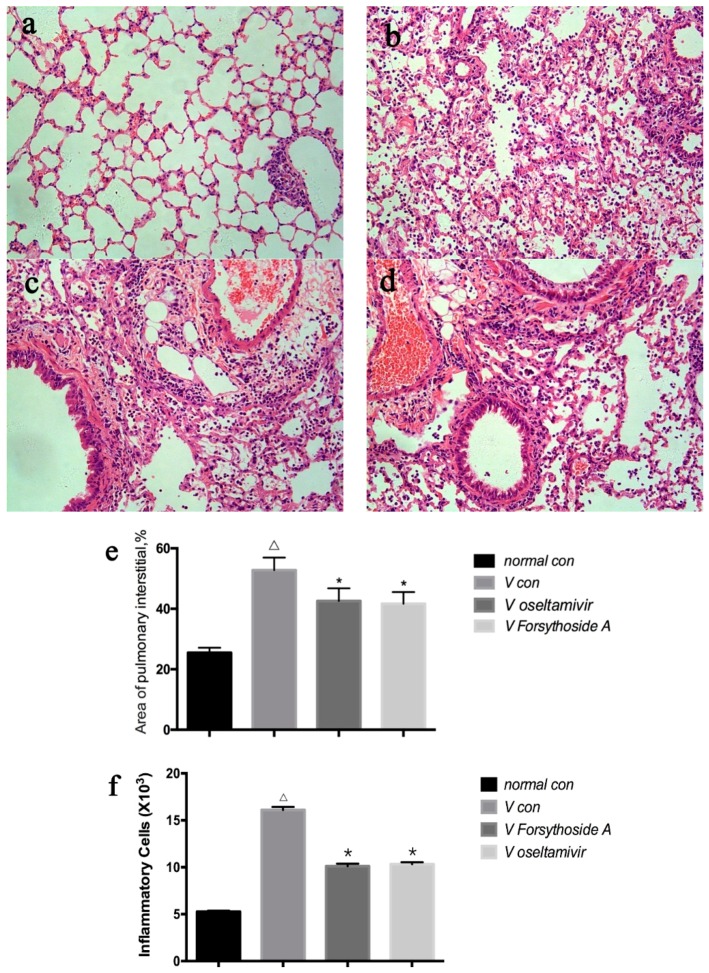
Effects of forsythoside A on the histological characterization in mice. (**a**–**d**) Representative hematoxylin and eosin staining histological sections of all the groups at day 6 ((**a**) *normal con*; (**b**) *V con*; (**c**) *V Forsythoside A*; (**d**) *V oseltamivir* group); (**e**,**f**) Changes in inflammatory cell counts and degree of edema in pulmonary interstitial. All images obtained at ×200 magnification. Δ *p* < 0.01 compared with *normal con*. * *p* < 0.01 compared with *V con*.

According to the inflammatory cell count and degree of edema in pulmonary interstitial results (Figure 3), the integrity of the structure of alveoli before FM1 infection was proved by pathological section examination.

The bronchial epithelium showed no external lesions or inflammatory cell infiltration (Figure 3a). After FM1 infection, diffuse damage could be seen in the alveoli, alveolar sacs, alveolar tubes, alveolar septa and bronchi. There was a large amount of lymphocyte infiltration in the pulmonary interstitium (Figure 3b). Compared with the *V con*, the pathological damage was alleviated in the *V oseltamivir* and *V FTA* groups. The alveolar interval was thinner in these two groups, the alveolar walls were diminished, infiltration of mononuclear cells in the walls of bronchioles decreased, but there was no distinct difference between *V oseltamivir* and *V FTA* after virus infection (Figure 3c,d). Total inflammatory cell count and degree of pulmonary interstitial edema increased after IAV infection (*p* < 0.01), reduced after Oseltamivir and FTA Treatment (*p* < 0.01) (Figure 3e,f).

### 2.3. Changes in Relative Expression of the Influenza A Virus Replication in Lung

**Figure 4:**
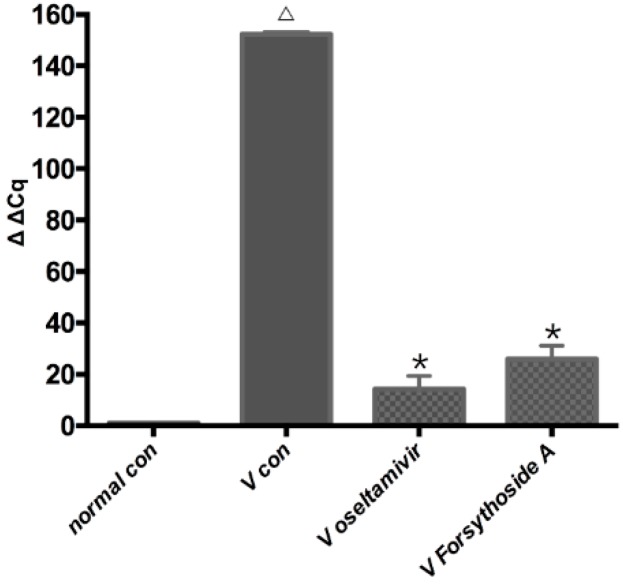
Effect of forsythoside A and oseltamivir on changing the relative expression levels of the IAV replication. Total RNA was isolated from the lung tissue. Statistics determined by two-part t test. Δ *p* < 0.01 compared with *normal con*. * *p* < 0.01 compared with *V con*.

There was no IAV replication in the normal control group. The virus replication increased rapidly after IAV infection. The virus infected animals in the *V con* group had a larger amount of replication than the *normal con* group (*p* < 0.01). Oseltamivir significantly reduced the virus replication compared with *V con* (*p* < 0.01). The replication of IAV in *V FTA* was higher, and had a statistical significant difference compared with the *V oseltamivir* group (*p* < 0.01) (Figure 4).

### 2.4. Relative mRNA Expression of TLR7, MyD88, TRAF6, IRAK4 and NF-κB

**Figure 5:**
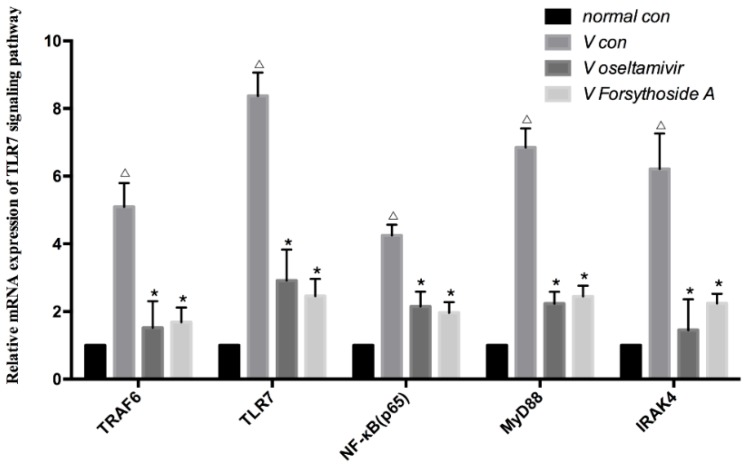
Relative mRNA relative expression of TLR7, MyD88, TRAF6, IRAK4 and NF-κB (p65) mRNA in the six groups (*n* = 12 each). Δ *p* < 0.01 compared with *normal con*. * *p* < 0.01 compared with *V con*.

As shown in Figure 5, the pulmonary immunocyte expression of TLR7, MyD88, IRAK4, TRAF6 and NF-κB mRNA in the TLR7 signaling pathways was significantly increased in *V con* (*p* < 0.01). 

Both oseltamivir and FTA treatment reduced mRNA expression in the TLR7 signaling pathway. Compared with the *V con* group, the mRNA in the TLR7 signaling pathway was down-regulated in the *V Oseltamivir* and *V FTA* groups (*p* < 0.01). Compared with the *V Oseltamivir* group, the difference of the *V FTA* one had no statistical significance (*p* > 0.01) (Figure 5). 

### 2.5. Relative Protein Expression of TLR7, MyD88 and NF-κB p65

**Figure 6:**
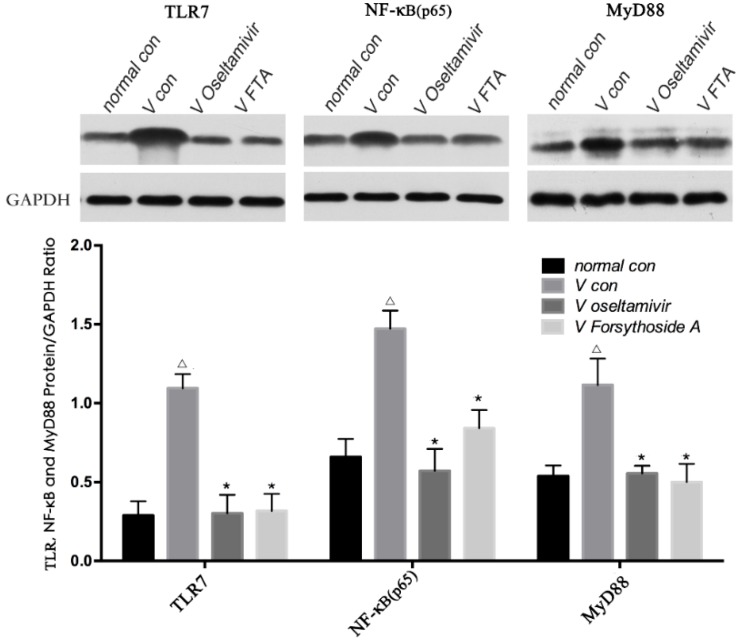
Relative protein expression of TLR7, NF-κB p65 and MyD88. Compared with the *normal con*, TLR7, MyD88 and NF-κB p65 protein expression was gradually enhanced in *V con*. Compared with the *V con*, the relative protein expression of TLR7, MyD88 and NF-κB p65 was down-regulated in *V Oseltamivir* and *V FTA*. Δ *p* < 0.01 compared with *normal con*. * *p* < 0.01 compared with *V con*.

According to the western blot results, TLR7, MyD88 and NF-κB p65 protein expression was gradually enhanced in *V con* compared with the *normal con* group. Compared with the *V con* group the relative protein expression of TLR7, MyD88 and NF-κB p65 was down-regulated in *V oseltamivir* and *V FTA* animals The western blot results were consistent with the RT-qPCR results (Figure 6).

### 2.6. Detection of Th1, Th2, Th17, and Treg Cells

**Figure 7:**
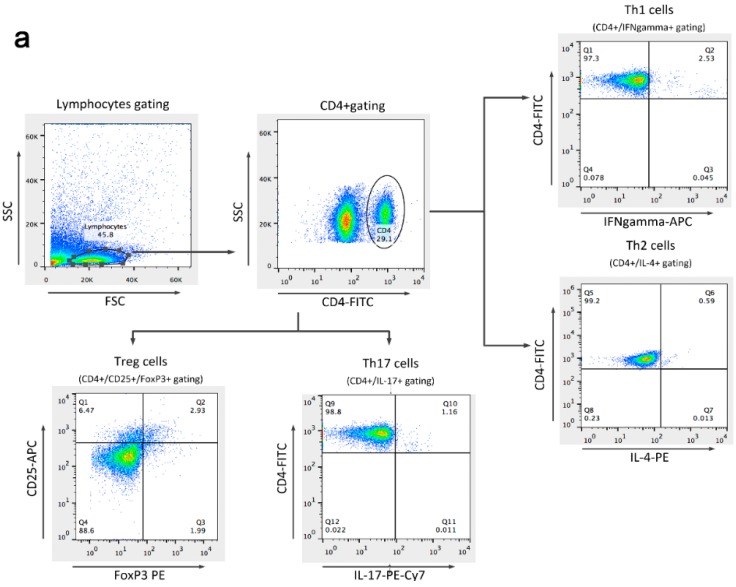

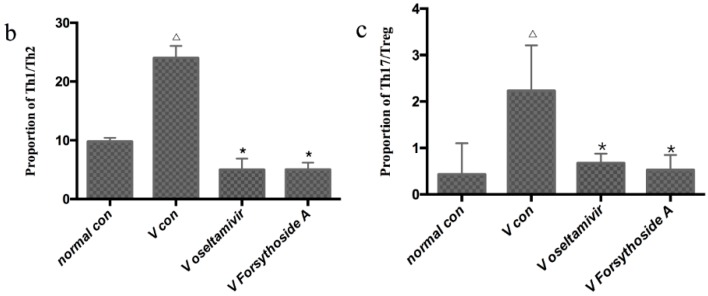
Flow cytometry was used to detect the proportion of the T cell subsets Th1/Th2 and Th17/Treg. (**a**) Flow cytometry analysis of CD4^+^ T cell subsets; (**b**,**c**) Changes in the proportion of Th1/Th2 cells and Th17/Treg cells. Δ *p* < 0.01 compared with *normal con*. * *p* < 0.01 compared with *V con*.

Flow cytometry was used to detect the proportion of the T cell subsets Th1/Th2 and Th17/Treg. These proportions increased after infection. The Th1/Th2 differentiated towards Th1, and the Th17/Treg cells differentiated towards Th17. The infected mice in the *V oseltamivir* and *V FTA* groups had lower proportions of these T cells subsets than infected mice in the normal group. FTA suppressed T cells from differentiating into Th1 or Th17 cells, and the proinflammatory roles of T cells were inhibited (Figure 7).

## 3. Discussion

Influenza A virus causes acute respiratory disease in mammals. Nearly 500,000 people die of IAV infection every year, primarily young children and the elderly [10]. IAV mainly spreads by coughing or sneezing of people with influenza [11]. The symptoms of IAV infection are fever, cough, sore throat, body aches, headache, chills and fatigue [12]. RT-PCR is a detection tool for the presumptive presence of IAV [12]. Oseltamivir is the orally-active prodrug of a carboxylate function, a specific inhibitor of influenza virus NA. Oseltamivir has been shown to be clinically active for the treatment and chemoprophylaxis of influenza and is currently approved for use worldwide [13]. However, oseltamivir causes nausea and vomiting and increases the risk of headaches and renal and psychiatric syndromes [14], side effects that are especially common in children [15]. Besides, oseltamivir-resistant IAV has circulated worldwide since the 2007–2008 influenza season [16]. There are several Chinese medicines having an anti-influenza A virus effect, including *Forsythia suspense* [17]. FTA, a natural molecule in this traditional Chinese herbal medicine, is one of the main phenylethanoid glycosides from *Forsythia suspense*. We believe it has a promising effect in the treatment of IAV infection.

Once a host is infected, the influenza virus elicits an innate response. TLR7 can recognize viral single-stranded RNA (ssRNA) then activate the downstream signaling molecules through the MyD88-dependent pathway [5]. TLRs can specifically recognize pathogen-associated molecular patterns (PAMPs) and transfer pathogen-related molecular signals into cells via transmembrane proteins. The pattern-recognition receptor (PRR) toll-like receptor 7 (TLR7) has been demonstrated to be a major sensor for the viral genome [4,18], and it serves as a crucial bridge between innate and adaptive immunity [5]. The activated TLR7 recruits MyD88 to endosomes, activating the TLR7 signaling pathway and then sending the signal to NF-κB through a series of cascade reaction [19,20,21]. The increasing concentration of pro-inflammatory cytokines and tumor necrosis factors [22], like TNF-alpha, IL-1, IL-17a, IL-17f and *etc.*, finally cause a huge excess of cytokines, known as a “cytokine storm”. The cytokine storm does more harm than good leading to immune pathologic dandification, particularly in lung. In different cytokines environment, CD4^+^ T cell differentiates into four lymphocyte subgroups [23,24], including Th1, Th2, Th17 and Tregs cells, The lymphocyte subsets work together to maintain immune equilibrium. Studies have shown that in the infectious environment endogenous TGF-β and inflammatory mediator IL-6 or IL-21 promote differentiation of Th17 to enhance inflammation [25,26]. TGF-β controlled function of effector cells in proper condition by Treg cells proliferation and maintaining their function [27,28]. Excessive inflammatory reaction may injure structure and function of body. It is an important subject of antiviral therapy to keep immunologic balance and avoid over excitation inflammatory response. 

It had been proved *in vitro* that extract of *Forsythia suspensa* has a protective effect on MDCK cells infected by H1N1 virus [29], but the mechanism whereby FTA improves the prognosis of IAV infection *in vivo* is poorly understood. In this study, the viral replication level RT-qPCR results showed that the influenza A virus replication was notably reduced in the *V oseltamivir* and *V FTA* groups. According to the RT-qPCR results of TLR7, MyD88, IRAK4, TRAF6 and NF-κB p65 mRNA, the expression of these TLR7 correlative mRNA were down-regulated after FTA treatment. The results indicated that FTA can inhibit influenza A virus replication leading to a down-regulation in TLR7 signaling pathway. TLR7, MyD88 and NF-κB p65 protein expression was consistent with the mRNA data. What’s more, hematoxylin & eosin staining results also confirmed the pathological damages of the *V FTA* were relieved compared with the *V con* group. FCM analysis on splenocytes exhibited a change in the Th1/Th2 ratio as well as the Th17/Treg ratio, which demonstrated FTA had an inhibitory action on the proinflammatory role of T cells.

In conclusion, this study demonstrated that the FTA had significant inhibitory effect against influenza A virus in mice. *Forsythia suspense* has been using for many centuries in humans, and no side-effect have been reported. However, we also know little about its pharmacological action. Our experiments therefore contribute to our knowledge of the pharmacology of FTA. Our study suggests FTA exerts an inhibitory effect on influenza A virus in mice, leads to down-regulating of TLR7 signaling pathway, controls influenza A virus infection and improves the prognosis of IAV infection. However, further experiments are needed to thoroughly clarify the potential of FTA in influenza virus infection treatment.

## 4. Materials and Methods

### 4.1. Animals

Forty-eight (half males and half females) specific pathogen free C57BL/6j mice weighing 20 ± 1 g were purchased from The Jackson Laboratory (The Jackson Laboratory, Sacramento, CA, USA). Mice were placed in a controlled environment of (23 ± 1 °C) and (50% ± 5%) relative humidity with free access to food and water for 14 days, under a 12 h light/dark cycle. Experiments were performed under the supervision and assessment of the Laboratory Animal Ethics Committee of Jinan University. All experimental procedures were performed in accordance with Guidelines on administration of Laboratory Animals, and were approved by the Animal Ethics Committee of Jinan University.

### 4.2. Grouping, Virus and Treatment

Mice were randomly divided into the following four groups, with 12 mice in each group: 

Group A: normal control group (*normal con*); Group B: IAV control group (*V con*); Group C: IAV+ oseltamivir treatment group (*V oseltamivir*, 0.78 mg/mL, 0.2 mL/mouse/day); Group D: IAV+ FTA treatment group (*V FTA*; 2μg/mL, 0.2 mL/mouse/day). 

Influenza A/FM1/1/47 (mouse adapted) used for all experiments was grown in the allantoic cavities of 10- to 11-day-old fertile chicken eggs for 2 days at 35 °C. Forsythoside A (>98% pure) was purchased from the Guangzhou Institute for Drug Control (Guangzhou, China). The mice in the *V con*, *V oseltamivir* and *V FTA* groups were fully anesthetized by inhalation of diethyl ether and then infected by intranasal application of 20% LD50 FM1 influenza virus suspension for 4 days (d1–d4). This procedure leads to upper and lower respiratory tract infection. Mice in *normal con* received saline after anesthesia by inhalation of diethyl ether. Treatments for mice in the *V oseltamivir* group and *V FTA* group were started at day 2 (d2–d5). 

### 4.3. Histopathological Examination 

Lung samples were obtained. Lung tissue (0.5 cm) was were removed for analysis, fixed with 4% paraformaldehyde, and then modified and trimmed by a blade. The appropriate lung part was selected and treated with the following steps: rinsing, dehydration, treatment by a transparent agent, immersing and other steps. After the sections were treated with drying, dewaxing, hydration and other steps, and stained with hematoxylin and eosin. The tissues were sectioned, and central tissue organization observed. The histopathology scoring methods for individual mice were the sum of two parameters, including the number of total inflammatory cells and degree of pulmonary interstitial edema [30,31]. The size of the microscopic field employed in the analysis was ×200. All analyses of slides were performed blind by a pathologist (Sizhi Wu) and quantified using image-pro-plus software (Media Cybernetics, Inc., Rockville, MD, USA).

### 4.4. RT-qPCR of TLR7, MyD88, TRAF6, IRAK4 and NF-κB p65 mRNA and Relative Expression of the Influenza A Virus in Lung

Lung samples were obtained. mRNA expression of TLR7, MyD88, TRAF6, IRAK4 and NF-κB was measured using quantitative real-time reverse transcriptase PCR (RT-qPCR), as well as IAV replication in lung. Total RNA was extracted by RNAiso Plus (No. 9108, TaKaRa, Kusatsu, Japan) according to the manufacturer’ s instructions. cDNA synthesis and real-time PCRs were carried out using the CFX Connect Real-Time PCR Detection system (BIO-RAD, Berkeley, CA, USA) with PrimeScript RT reagent kits (RR047A, TaKaRa) and SYBR Premix EX Taq II (RR820A, TaKaRa) according to the manufacturer’ s instructions. Primers were synthesized by Generay Biotech Co. (Shanghai, China). All primers for RT-qPCR are presented in Table 1. After RT-qPCR, analysis of the relative gene expression levels was performed using the 2^−ΔΔCT^ method. Each sample was measured three times and averaged. Gene expression in the *V oseltamivir* and *V FTA* groups was expressed relative to the *V con* group.

### 4.5. Western Blot for TLR7, MyD88 and NF-κB p65

Proteins were extracted from the lung tissues and then quantified using BCA protein assay kits (Wuhan Goodbio Technology Co., Ltd., Wuhan, China). An equal amount of protein (20 μg/lane) was fractionated using an electrophoresis system (BIO-RAD) on 10% and 15% polyacrylamide gels and then transferred to PVDF membranes (Millipore, Darmstadt, Germany). Membranes were respectively incubated with antibodies at an appropriate dilution Glyceraldehyde-3-phosphate dehydrogenase (GAPDH), NF-κB p65 (Santa Cruz Biotechnology, Dallas, TX, USA), TLR7 and MyD88 (Cell Signaling Technology, MA, USA) at 4 °C overnight. Membranes were washed and incubated with secondary antibody for 30 min at 37 °C. Protein bands were detected using an electrochemiluminescence kit according to the manufacturer’ s instructions and analysed using GE Image Quant LAS 4000 mini imaging analysis software (BIO-RAD).

### 4.6. Immunofluorescence Labeling and Flow Cytometry

PBMCs were isolated from the spleen by lymphocyte separation medium (TBDscience Co., Tianjin, China) according to the manufacturer’s instructions. Different subsets of T cells were evaluated by flow cytometry. All anti-mouse-specific Abs used in this study were obtained from eBioscience (San Diego, CA, USA). PBMCs were stimulated with phorbol myristate acetate (PMA, 25 ng/mL, MultiSciences Biotech Co., Ltd., Hangzhou, China) and ionomycin (1 μg/mL, MultiSciences Biotech Co.) in the presence of FC Receptor Blocker (MultiSciences Biotech Co.) for 4 h. The cells were washed and then fixed/permeabilized in the eBioscience fixation/permeabilization and permeabilization buffers and stained with anti-CD4-FITC, anti-CD25-APC, anti-IL-4-PE, anti- IFN gamma-APC, anti-IL-17-PE-Cyanine7, and anti-Foxp3-PE. Appropriate isotype controls were performed. Flow cytometry was performed on a BD FACS Calibur flow cytometer (BD Biosciences, Franklin Lakes, NJ, USA) and analyzed by using FCS Express3 software (De Novo, Kiev, Ukraine).

### 4.7. Statistical Analysis

Statistical analyses were carried out using SPSS 22 for Mac (IBM Software, New York, NY, USA). All data are presented as mean ± SD. Groups were compared using one-way ANOVA, followed by *post-hoc* Student-Newman-Keuls tests. The values of protein band density obtained from gel analysis and band densitometry were calculated. These values were expressed as TLR7, NF-κB or MyD88/GAPDH ratio for each sample. The averages for different groups were compared using ANOVA followed by the Newman-Keuls test. A p value of <0.01 was considered to be statistically significant.

## Figures and Tables

**Table 1:** Primers used for RT-qPCR analysis.

Gene	Primer	Sequence
GAPDH	Forward primer	5′-TGATGACATCAAGAAGGTGGTGAAG-3′
	Reverse primer	5′-TCCTTGGAGGCCATGTAGGCCAT-3′
TLR7	Forward primer	5′-GGGTCCAAAGCCAATGTG-3′
	Reverse primer	5′-TGTTAGATTCTCCTTCGTGATG-3′
MyD88	Forward primer	5′-CGATTATCTACAGAGCAAGGAATG-3′
	Reverse primer	5′-ATAGTGATGAACCGCAGGATAC-3′
TRAF6	Forward primer	5′-TTGGAGAGTCGCCTAGTAAG-3′
	Reverse primer	5′-GTTACACTGCTGTGCTTCC-3′
IRAK4	Forward primer	5′-CATCGTGGCGGTGAAGAAG-3′
	Reverse primer	5′-AGCATACACTAAGCACAGGTTG-3′
NF-κB p65	Forward primer	5′-ATTCTGACCTTGCCTATCTAC-3′
	Reverse primer	5′-TCCAGTCTCCGAGTGAAG-3′
Virus Replication	Forward primer	5′-GACCAATCCTGTCACCTCTGAC-3′
	Reverse primer	5′-GGGCATTTGGACAAACGTCTACG-3′

RT-qPCR, quantitative real-time polymerase chain reaction.
